# Scat‐Tered Evidence. Understanding the Diet of Forest‐Associated Mammalian Mesopredators in a UK Peatland Ecosystem

**DOI:** 10.1002/ece3.71961

**Published:** 2025-08-14

**Authors:** R. McHenry, L. J. Mitchell, J. Smart, R. Andersen

**Affiliations:** ^1^ Environmental Research Institute University of the Highlands and Islands Inverness UK; ^2^ RSPB Centre for Conservation Science The Lodge, Sandy Bedfordshire UK; ^3^ School of Biological Science University of East Anglia Norwich UK

**Keywords:** DNA metabarcoding, ground‐nesting birds, peatland, predation, predator diet, scat identification, waders

## Abstract

Peatland ecosystems and the unique biodiversity they support are under risk from multiple stressors, including changes in species interactions. Land use changes that lead to increases in the density and activity of mammalian mesopredators such as red fox ( *Vulpes vulpes* ) and pine marten ( *Martes martes* ) could be particularly detrimental to vulnerable peatland species such as wading birds (*Chardiiformes*). However, our understanding of predator–prey interactions in the context of land use change remains limited, because most published evidence is correlative. In contrast, DNA metabarcoding of scats can reliably identify both the host and the prey consumed, thereby clarifying the role of consumption in species interactions. In this study, we collected scats from areas of open peatland and non‐native forestry on peatland across the Forsinard Flows Nature Reserve, part of The Flow Country UNESCO World Heritage Site in Scotland. We focused our collections during the wader breeding season of 2023. Following DNA extraction and metabarcoding, we found that diets of foxes and pine marten were made up of small mammals (e.g., *Rodentia* and *Soricidae*), non‐wader bird species, and frogs. By frequency of occurrence, birds formed a substantial part of the pine marten diet (50%), while red deer carrion and pine marten (or their scat) were key food resources for foxes (46% and 50% respectively). Surprisingly, wading birds were absent from our samples, challenging the assumption that forest‐associated mammalian predators actually prey on waders in adjacent peatland. DNA metabarcoding may be crucial to understanding the trophic and non‐trophic interactions that govern recruitment and habitat use of vulnerable native species in remote and complex landscapes.

## Graphical abstract

DNA metabarcoding of predator scat can provide direct evidence of trophic interactions in a landscape that may refute prior indirect evidence based on correlative data.

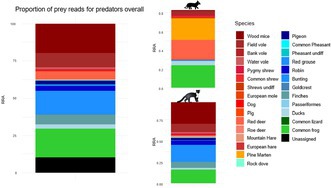

## 1 Introduction

Anthropogenically modified landscapes have altered predator–prey dynamics (e.g., Cimatti et al. 2021; DeMars and Boutin 2018; Grande et al. 2018; Reif et al. 2023); yet the effects of these alterations on the food web and wider ecosystem functioning are poorly understood. Across Europe, changes such as the cultivation of farmland, deforestation, and conversely, planting of forests have created a landscape mosaic that favors adaptable generalists such as the red fox ( *Vulpes vulpes* ) and corvid species, enabling them to persist in landscapes they did not previously inhabit (Balharry 1993).

This is exemplified in the Flow Country of Caithness and Sutherland (58°21′12.4″N 3°54′19.0″W), the largest area of contiguous blanket bog in the world. It has recently been designated a UNESCO World Heritage site and a major breeding area for protected waders (*Charadriiformes*). From the 1960s to the 1980s, approximately 67,000 ha (16%) of the Flow Country peatlands were drained and afforested with non‐native conifers (Lindsay et al. 1988; Stroud et al. 1988). The habitat was then further fragmented by the construction of forest roads. The forests brought with them an increase in previously scarce, forest‐associated mesopredators such as red fox ( *Vulpes vulpes* ) and pine marten ( *Martes martes* ) which had previously been confined to wooded straths (fertile riparian valleys) and farmland (Balharry 1993; GWCT (Game and Wildlife Conservation Trust) 2024; Scottish Natural Heritage 2013; Lloyd 1980; Pereboom et al. 2008; Roos et al. 2018; Sainsbury et al. 2019).

Alongside these land‐use changes, the extirpation and decline of apex predators, for example, Lynx ( *Lynx lynx* ), wolves ( *Canis lupus* ) and white‐tailed eagles ( *Haliaeetus albicilla* ) combined with the persecution of smaller raptors such as hen harriers ( *Circus cyaneus* ) and buzzards ( *Buteo buteo* ), has enabled ‘mesopredator release’. This process, whereby mid‐size carnivores (‘mesopredators’) are released from top‐down control, causes rapid population growth and range expansion (Soulé et al. 1988; Pasanen‐Mortensen et al. 2013; Prugh et al. 2009). These generalist predators have also further benefited from reduced persecution because culling for the protection of game species populations (gamekeeping) has declined, and conservation legislation has been strengthened (e.g., protected statuses for pine marten and badger ( *Meles meles* ) (Reason et al. 1993)). Likewise, inflated red grouse (*Lagopus scotica*) and red deer ( *Cervus elaphus* ) populations as a result of land management, supplementary feeding, and lack of apex predators provide substantial supplementary prey and carrion resources for mesopredators (Pringle et al. 2019; GWCT (Game and Wildlife Conservation Trust) 2024).

In parallel with mesopredator increases, persistent declines of European birds, particularly ground nesting species, such as waders, have occurred (McMahon et al. 2024). In the Flow Country, forestry plantations on the peatland have affected wader distribution and recruitment (Hancock et al. 2009, 2020; Stroud et al. 1988; Wilson et al. 2014), attributed in part to predation by mammalian mesopredators (McMahon et al. 2020). So far, in other systems, researchers have sought to understand the relationship between wader declines and mesopredators by looking at predator activity near nesting sites through camera trapping (e.g., Kaasiku et al. 2022; Krüger et al. 2018; Laux et al. 2022). However, not only activity does not directly imply predation, but camera traps often fail to capture fast moving and nocturnal mesopredators, so the realistic level of activity is often not known (McHenry et al. 2025).

A correlative study by Hancock et al. (2020) related scat counts from fox and pine marten to breeding success and presence/absence of waders in the Flow Country. Scat counts were 2.9 times higher within 700 m of forestry than on open bog, and the authors related this to the 700 m forestry edge effect on waders described by Wilson et al. (2014). However, while scat counts can provide some information on the presence of a mesopredator, they do not necessarily reveal the nature of the interactions. Moreover, as the direct identification of scat origin is prone to inaccuracy (Davison et al. 2002; Harrington et al. 2009; Lonsinger et al. 2015; Monterroso et al. 2013; Morin et al. 2016), scat counts alone cannot be relied upon to determine species‐specific presence; rather, they indicate general predator activity and presence in a landscape. To our knowledge, so far, no direct evidence of widespread predation of waders by mesopredators in Scottish peatlands has been provided. Discerning if predators are suppressing wader populations through direct predation or indirectly through some other mechanism is crucial to our understanding, quantification, and management of their impact on protected and endangered species.

DNA metabarcoding (from hereon, metabarcoding) is increasingly being used to analyse the diet of wildlife (Buzan et al. 2024; Shippley et al. 2025; Tuomikoski et al. 2024, Waggershauser et al. 2022; Youngmann et al. 2023). Metabarcoding is a comprehensive, non‐invasive sampling method to analyse the prey taxa consumed by predators, while simultaneously confirming the identity of the predator itself. Unlike macroanalysis of prey remains, molecular approaches can identify the prey even where only soft parts are consumed, for example, the interior of bird eggs (Tuomikoski et al. 2024). Furthermore, as well as a comprehensive list of prey taxa, metabarcoding can provide researchers with clues as to the relative amount of prey being consumed via ‘relative read abundance’ or RRA, which refers to the use of sequence counts to weight taxa present in samples through relative proportions. However, this is an uncertain relationship due to differential rates of tissue digestion, DNA degradation rates, and primer amplification biases (Deagle et al. 2019) and should not be misinterpreted as absolute abundance.

Evidence from DNA metabarcoding can also indicate whether predators opportunistically predate certain species while subsisting on others (e.g., Hacker et al. 2021; Hardy et al. 2024) and can reveal dietary overlap between predator species across habitats (e.g., Hacker et al. 2022; Havmøller et al. 2021; Shi et al. 2021), providing insight into competition for prey with vulnerable predator species for example, golden eagle ( *Aquila chrysaetos* ), white‐tailed eagle ( *Haliaeetus Albicilla* ) and hen harrier ( *Circus cyaneus* ), many of which are reestablishing populations in the Scottish Highlands, including in the Flow Country (Hughes et al. 2024).

In this study, we used metabarcoding of mesopredator scats to investigate the interaction between pine marten and red foxes with forestry and peatland dwelling prey species. Our objectives were: (1) to confirm the predator species from each collected scat; (2) to investigate the prey taxa of pine marten and red fox in the flow country peatland‐forest mosaic, with the aim of assessing the actual impact of these mesopredators on waders; and (3) to identify ecological/trophic interactions in these landscapes.

We expected to confirm that scats from both predator species had been collected, but that some would have been misidentified in the field. We also expected to find a wide variety of prey taxa, of which birds from both the peatland and the forestry would make up a significant portion of reads or species consumed, given the lack of mammal prey in the peatland interior, and timing of scat collection within the bird breeding season.

## 2 Methods

### 2.1 Study Area

**FIGURE 1:**
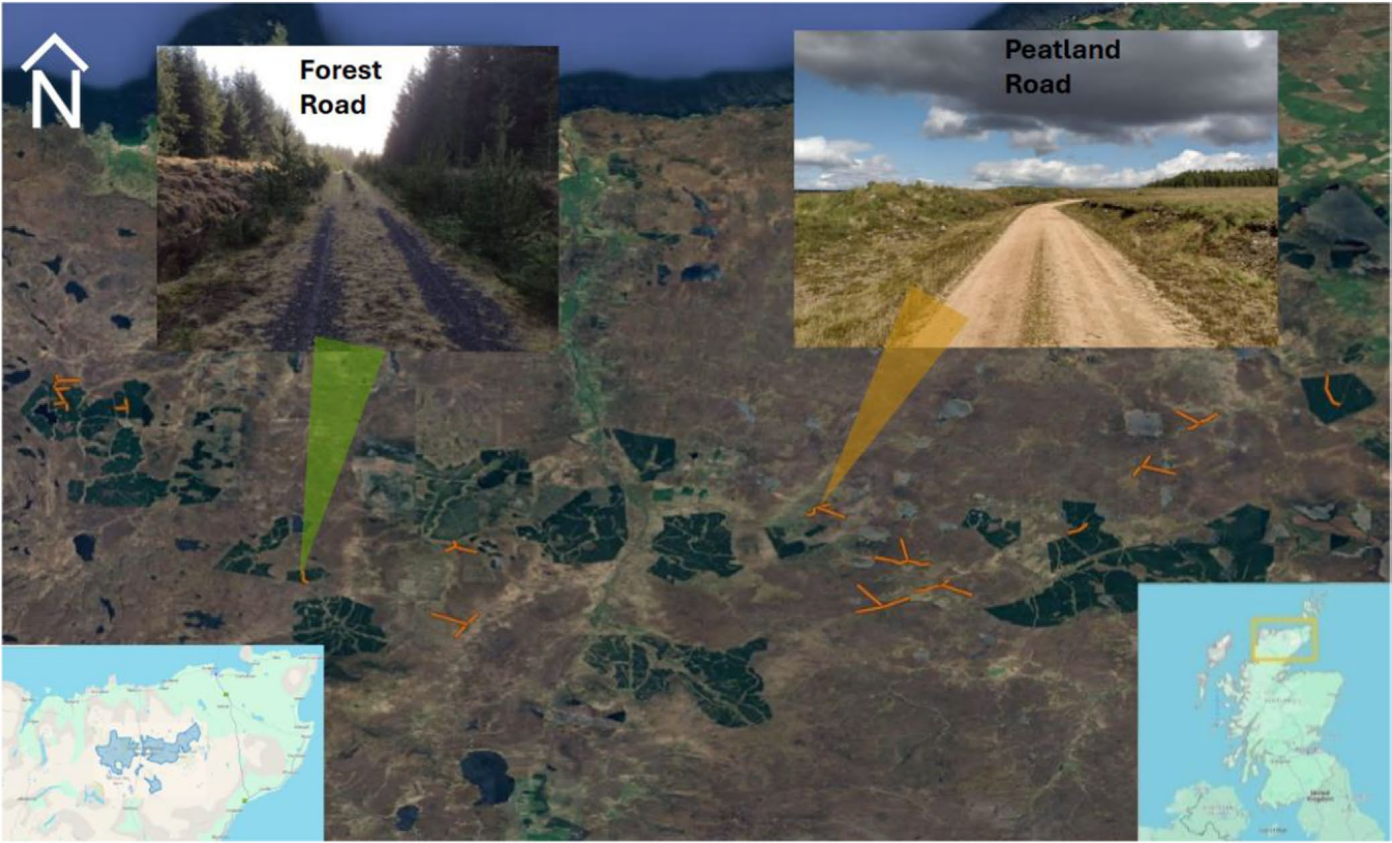
An example of one peatland road (yellow) and forestry road (green) where scats were collected along and adjacent to, the Flow Country National Nature Reserve (bottom left) Within the wider Flow Country (bottom right) Scotland.

We undertook scat counts within the Royal Society for the Protection of Birds' Forsinard Flows Nature Reserve, part of the wider Flow Country, covering an area of 4,000 km^2^, encompassing a mix of intact blanket bog, areas under restoration management (not included in this study) and conifer plantations (Figure 1). The area has very little natural shelter and relatively low densities of native ground predators (e.g., 0.5–1.5 fox/km^2^, Webbon et al. 2004, unpublished data). The naturally treeless peatland habitat was fragmented by plantations of non‐native conifers in the 1970s–80s, generally Lodgepole pine ( *Pinus contorta* ) and Sitka spruce ( *Picea sitchensis* ). Those plantations, according to camera trap and scat count data (Hancock et al. 2020) contain higher densities of mammalian mesopredators (up to 25 times more in one forestry site vs. another peatland site in the same period. (McHenry et al., *in prep*)). Both the peatland and the forestry are intersected by forestry tracks.

### 2.2 Sampling Protocol

As part of a wider study, we collected scats seasonally (4 counts/year) from 27,500 m transects walked twice per count along sections of peatland road (*n* = 9), adjacent open peatland (*n* = 9), forestry roads (*n* = 6) and forestry itself (*n* = 3) from spring 2022 until winter 2023 (168, transect seasons (189,000 m total transect walked)). We collected all scats that were identified as either pine marten or fox by appearance, except when they were degraded to the point that their distinct form and colour was lost and thus were no longer conclusively fox or pine marten. We collected scats of the same species, less than 1 m apart as one sample so as to reduce the chance of double counting. We then froze scats in a −20°C freezer for a maximum of 10 months and then in a −80°C freezer thereafter once that facility became available. For this study, we chose a total of 50 scat samples from across the study area and timeframe for analysis from a total of 221 according to the following hierarchy: (1) collected during the bird breeding season and in a high quality of preservation (i.e., scat still moist and unbroken on collection, *n* = 20); (2) collected during the bird breeding season and in medium quality of preservation (i.e., scat is unbroken but no longer moist, (*n* = 18)); (3) from anytime across the non‐breeding season but in a high quality of preservation (*n* = 12). Samples that were preserved solely in the −80°C freezer were prioritized within this hierarchy. We did not use low quality scats (scat is broken and no longer moist or fully desiccated) for molecular analysis.

Hence, we collected most scats (76%) during the wader breeding season (mid‐April to mid‐June in the UK, (Balmer et al. 2013)), which coincides with increased reptile and amphibian activity (McInerny 2017) and overlapped with the breeding period of small mammals such as shrews (*Soricidae*) and field voles (*Microtus*) (Gębczyński 1965; Myllymäki 1977).

### 2.3 DNA Extraction and Metabarcoding

We subsampled 50 mg of each scat for DNA extraction and metabarcoding. Due to the large portion of cells in the scats that may have undergone lysis during digestion, and may have adsorbed to the soil/mineral substrate that had adhered to the scat, samples were first mixed with equal parts sodium lysis buffer for 20–30 min. This is an effective method for extracting extracellular DNA because phosphate competes with DNA for adsorption (Taberlet et al. 2012). We used a NucleoSpin Soil Kit to extract a final elution volume of 100 μL following the manufacturer's instructions (Macherey‐Nagel, Germany). We used vertebrate‐specific primers that target a 97 bp fragment of the mitochondrial 12S ribosomal RNA (rRNA) region in fish (Riaz et al. 2011) to PCR‐amplify DNA extracts. Primers were tagged for the present study to include Multiplex Identification (MID) tags, heterogeneity spacers (a nucleotide region, independent of the binding specificity or adapter regions required for Illumina sequencing, which serves to increase base diversity within each sequencing cycle by offsetting the targeted nucleotides), sequencing primers, and pre‐adapters (Fadrosh et al. 2014; Jensen et al. 2019; Kitson et al. 2019).

### 2.4 Data Analysis

After sequencing, we removed redundant sequences by clustering at 100% read identity and length (–derep_fulllength) in VSEARCH (Rognes et al. 2016). We omitted clusters represented by less than three sequences from further processing. We further clustered reads (–cluster_unoise) to remove redundancies due to sequencing errors (retaining all cluster sizes). We screened retained sequences for chimeric sequences with VSEARCH (–uchime3_denovo). We used BLAST (Zhang et al. 2020) to compare the final clustered, non‐redundant query sequences against a curated UK vertebrate reference database (Harper et al. 2018). We used a custom majority lowest common ancestor (MCLA) approach to assign taxonomic identity based on the top 2% query BLAST hit bit‐scores, with at least 90% query coverage and a minimum identity certainty of 98%. Of these filtered hits, 80% of unique taxonomic lineages therein had to agree in descending taxonomic rank (domain, phylum, class, order, family, genus, species) for it to be assigned a taxonomic identity. If a query had a single BLAST hit, we assigned it directly to this taxon only if it met all MLCA criteria. We assigned read counts to each taxonomic identity calculated from query cluster sizes. Lowest taxonomic rank was to species, and assignments higher than order were classed as unassigned.

Following taxonomic assignment, we applied a noise threshold of 0.1% of total reads per sample to remove low frequency reads (Hänfling et al. 2016). We assigned most reads to the species level, but because the molecular marker used cannot distinguish certain species reliably, especially avifauna for example, Ducks (Anatidae), we assigned the reads belonging to these species to the next possible higher taxonomic level. Reads assigned to positive controls, those which could not be assigned to any taxon, and samples with no taxonomically assignable reads were removed from the data set.

Lastly, we calculated frequency of occurrence (FOO) (the number of samples that contain a given food item, most often expressed as a percent (%FOO) (Deagle et al. 2019)) and relative read abundance (RRA) the proportion of DNA sequences belonging to a specific taxa relative to the total number of sequences in a dataset (Deagle et al. 2019) for each prey species and genera where relevant, after subtracting human contamination and host species. We set a minimum read threshold of 100; taxa that fell below this threshold were removed from analysis (see Appendix S1 for more detailed lab protocol).

## 3 Results

### 3.1 Confirming Predator Identity

Host species were generally the species with the highest number of reads per sample. In two samples there was insufficient DNA present to confidently identify either host or prey species; we removed these from the analysis. Of the 48 analysed samples, 50% were fox and 50% pine marten. DNA analysis revealed that eight of the pine marten scats (33.3%) were misidentified as fox, and six of the fox scats (25%) were misidentified as pine marten in the field. No scats were from a non‐target host.

### 3.2 Investigating Predator Diet—Frequency of Occurrence

**FIGURE 2:**
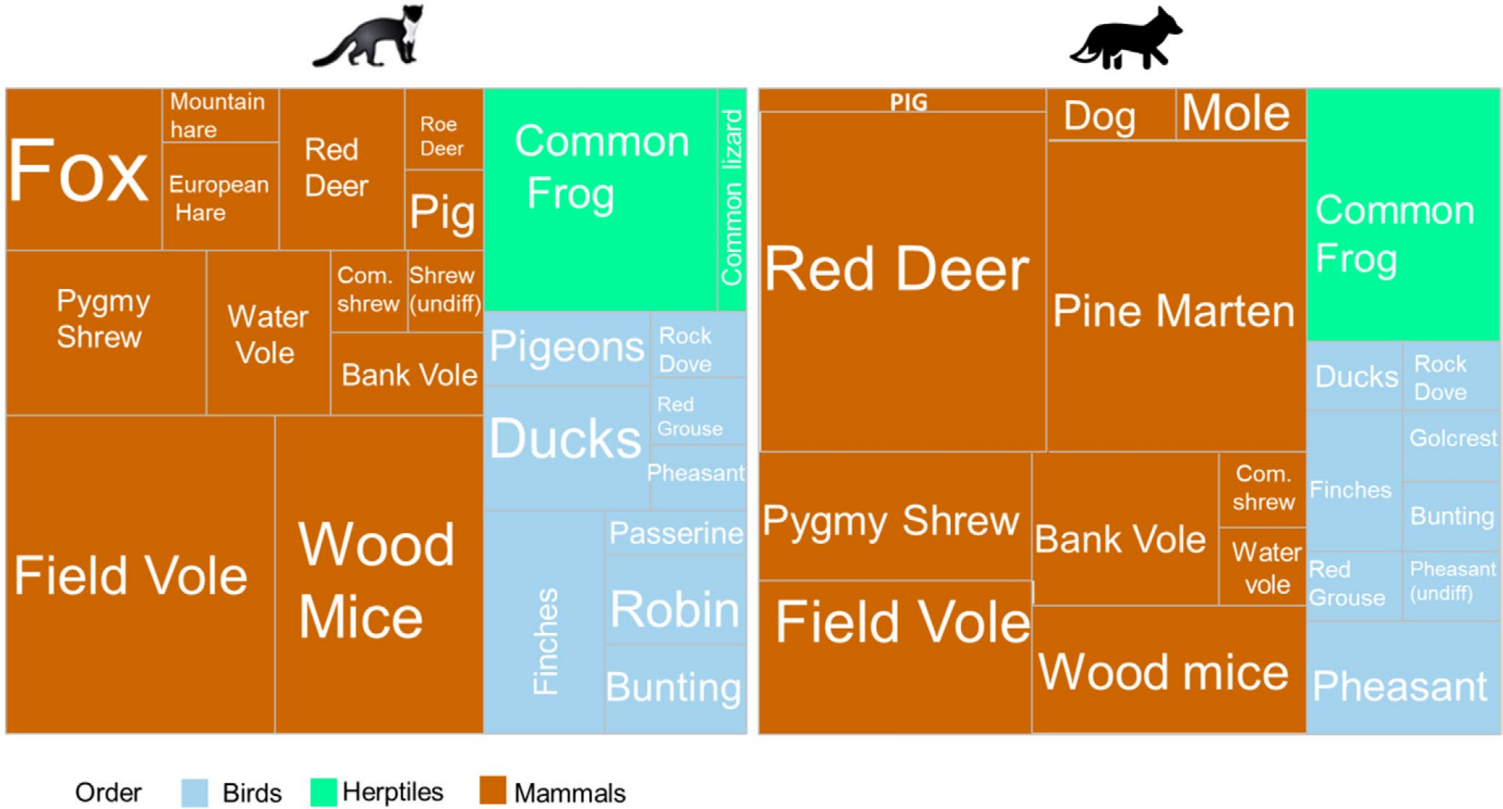
Prey taxa by frequency of occurrence for pine marten and fox. The size of the square is proportionate to the percentage of samples containing that species.

By FOO, the most common species when looking at both predators combined was field vole ( *Microtus agrestis* ; 40**%**) based on reads assignable to species. As a genus, wood mice (*Apodemus*) were the most frequently consumed prey (58%), and if amalgamated with voles and shrews to create a small‐mammal group, they make up the majority of prey (63%). This second most consumed species was the common frog ( *Rana temporaria* ; 27%). Other species found included red deer ( *Cervus elaphus* ) (27%), Lagomorphs (6%), common lizard (*Zootoca vivipara*) (2%) (Figure 2).

Altogether, birds were present in 33% of samples and included finches (*Fringillidae sp*), common pheasant ( *Phasianus colchicus* ), buntings (*Emberiza sp*), ducks (*Anatidae sp*), pigeons (*Columba sp*) and grouse (*Lagopus sp*). Passerines as a group were found in 19**%** of the samples. With the exception of grouse and ducks, most of the bird species found are not specific peatland species that are instead associated with forestry, lakes, and woodlands. The ratio of woodland: open habitat species was 50:50 for fox samples and 70:30 for pine marten. We found no evidence of consumption of waders in our study, except in a single sample with 92 reads for woodcock ( *Scolopax rusticola* ), this was below the read threshold (100).

Pine marten had a more varied diet with a higher prey richness per scat than fox; however, overall species richness across samples was very similar, with 24 species present across pine marten samples and 22 across fox samples. Rodents were present in 60% of pine marten scats compared to 33% of fox scat, with field vole and wood mice far more common in pine marten scats than fox (64% vs. 21% and 50% vs. 13%, respectively (Figure 2)). Birds were uncommon in fox scats (12.5%) but present in half (50%) of pine marten scats. Meanwhile, red deer was present in nearly half (46%) of fox scats but only 8% of pine marten scats. Finally, pine marten DNA was present in 50% of fox scats, while fox DNA was present in 16% of pine marten scats.

**FIGURE 3:**
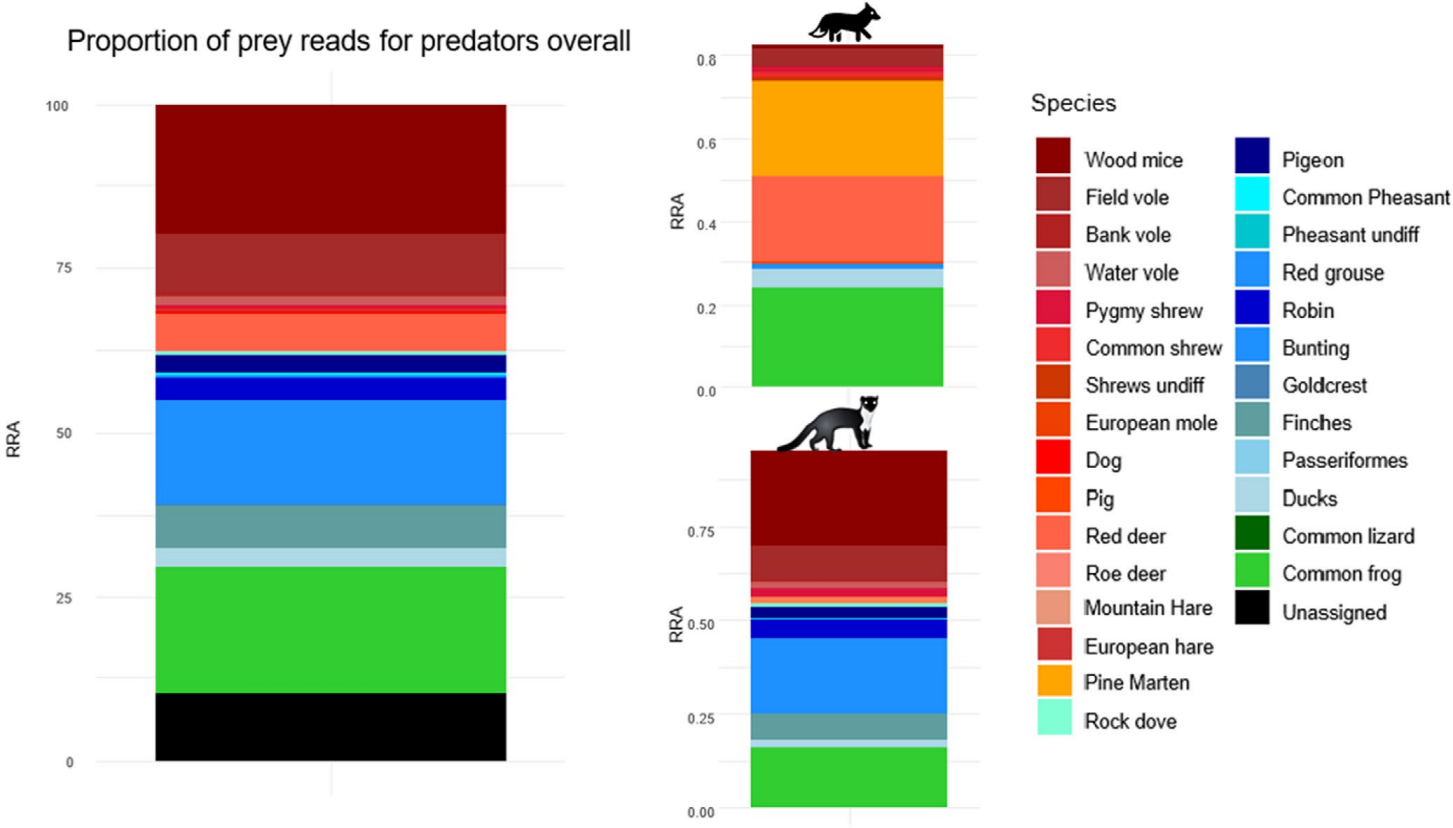
Prey taxa by relative read abundance (RRA) for both predators and for pine marten and fox individually. The size of each segment is proportional to the portion of total reads for that species.

In terms of RRA, small mammals, particularly wood mice, made up 40% total reads for pine marten, followed by birds which form another 40% of pine marten diet, including 20% solely for bunting species. Frogs ( *Rana temporaria* ) also contributed to pine marten diet (16%). When looking at fox scats, frogs accounted for a greater proportion of RRA (24%), followed by pine marten (23%) and red deer (21%). Contrary to scats from pine marten, reads from small mammals (8.5%) and birds (5.4%) were much less prevalent in fox scats. (Figure 3).

## 4 Discussion

### 4.1 Fox and Pine Marten Prey Taxa in the Flow Country

We expected that the scats collected during the wader breeding season would contain DNA from wader species, based on assumptions derived from correlative studies (Hancock et al. 2009; Hancock et al. 2020; McMahon et al. 2020; Wilson et al. 2014), and the belief that forest‐associated mesopredators are a major cause in the decline of nesting waders in peatlands (Hancock et al. 2020, 2009; Wilson et al. 2014). However, we found no evidence of any wader consumption.

Detected bird DNA was largely from woodland species and mainly appeared in pine marten scats. In contrast, small mammals dominated the diet of both predators alongside a large proportion of frogs. Fox scats also contained a high proportion of red deer DNA; here, as predation of adults can be ruled out, this suggests scavenging on abundant deer carcasses across the Flow Country as a major feeding strategy (Bassi et al. 2018; Wikenros et al. 2024). Although some deer DNA in both predator scats may stem from environmental contamination, the levels in fox samples exceed what could be explained by contamination alone. Deer carcasses are likely abundant in the Flow Country due to train strikes, disease, and harsh weather; as are grallochs (the inner organs and bones left behind after deer culling or poaching). Given the size of deer, even limited scavenging within 72 h could yield substantial DNA in scat samples.

Pine marten scats had greater species richness than fox scats, consistent with previous studies (Caryl et al. 2012; Grabham et al. 2019; Twining et al. 2019); and given their ecological position as woodland specialists, they may have had access to the relatively high number of bird and small mammal species present in forestry compared to peatland, particularly in forestry canopies compared to the forest floor.

By contrast, foxes are dietary and habitat generalists. As facultative carnivores, they are more likely to scavenge carcasses, exploit anthropogenic food sources, and engage in coprophagia (Castañeda et al. 2020; Waggershauser et al. 2022; Wikenross et al., 2024). As poor climbers, foxes are unable to access birds and nests in the canopy.

Of the birds consumed, several, for example, red grouse ( *Lagopus lagopus* ), meadow pipit ( *Anthus pratensis* ) are associated with open habitat, including peatlands, or are associated with forestry, e.g., finches (*Fringillidae*) and robin ( *Erithacus rubecula* ) (Balmer et al. 2013). It was expected that woodland birds would make up a large portion of the predator diet given that pine marten are specialist arboreal predators and because foxes are more associated with forestry than peatland. However, the ratio of forest to open habitat‐associated species was greater than expected given the relatively small patches of forest and the timing of scat collection with expected availability of wader eggs and chicks. There exists some limited direct evidence of wader predation by mesopredators in similar open habitats in Europe in the current literature (Carpio et al. 2016; Draycott et al. 2008; Eglington et al. 2009; Mason et al. 2018; Mori et al. 2021). However, none of these studies are in peatlands, nor do they focus on waders under threat in the Flow Country, for example, golden plover or dunlin. Only two (Eglington et al. 2009 and Mason et al. 2017) were carried out in the UK.

Absence of wader DNA in predator scats may have been due to limited spatial overlap of waders with fox and pine marten territories. Waders typically avoid forestry and have reduced nest densities within several 100 m of forest edges (Kaasiku et al. 2022; Pálsdóttir et al. 2022; Wilson et al. 2014). Since all peatland sites in our study lie 830 m to 3 km from forestry, edge effects may reduce the presence of species like dunlin ( *Calidris alpina* ) and golden plover ( *Pluvialis apricaria* ). However, greenshank ( *Tringa nebularia* ) and snipe ( *Gallinago gallinago* ) appear less affected by forestry (Hughes et al. 2024; Nethersole‐Thompson and Nethersole‐Thompson 1979; Wilson et al. 2014). Although forest‐to‐bog restoration has occurred in the area, its impact on wader recruitment remains uncertain (Ward 2024). Overall, waders may not be targeted simply because they are absent from predator foraging areas.

The common occurrence of frog DNA may be due to the season in which the scats were collected. May and June often coincide with the emergence of large quantities of juvenile frogs (Vos et al. 2007). Frogs may spawn in road ditches alongside forestry or open bog (DeMaynadier and Hunter Jr 1995), and we hypothesize that predators are consuming frogs on the peatland or peatland edges, either incidentally or deliberately to take advantage of this transient abundance of prey.

### 4.2 Predator Identification

Altogether, nearly one in three scats was misidentified. This level of accuracy is typical of most comparisons between visual and DNA identification and may have benefited from there being only one scat identifier (Davison et al. 2002; Harrington et al. 2009; Lonsinger et al. 2015; Monterroso et al. 2013; Morin et al. 2016). This underlines the need for DNA confirmation of host identity in studies where species identity is key to analyses and the resulting scientific conclusions or policy recommendations, or the need for a level of caution when DNA confirmation is not possible.

### 4.3 Ecological/Trophic Interactions in the Flow Country Peatland‐Forest Mosaic

We found pine marten DNA in fox scats and, to a lesser extent, fox DNA in pine marten scats. In some samples, the number of pine marten and fox reads was almost equal, more than could be attributed to contamination alone, suggesting direct consumption. Overmarking of scats (deliberate deposition of scats over another individual's scat) was not uncommon during our collections and is documented in the literature (Apps et al. 2019; Blizard and Perry 1979; De Monte and Roeder 1990), although generally, where two scats were found together, we tried to sample a non‐overlapping portion. There are also recorded cases of interspecific predation between pine marten and fox in both directions (e.g., Brzeziński et al. 2014; Lindström et al. 1995; Waggershauser et al. 2021), especially where cubs are involved, which would have been present during the survey period. Further, a study by Waggershauser et al. (2022) has shown the widespread consumption of dog ( *Canis lupus familiaris* ) faeces by foxes in the Cairngorms, UK, and indeed, dog DNA was recorded in one fox sample in this study. Although pine marten scats are of substantially lower caloric value than dog scats (Waggershauser, personal correspondence), they could instil microfaunal or nutritional benefits. Thus, interspecific coprophagia between fox and pine marten in both directions cannot be ruled out and underlines the need for verification of metabarcoding data with macro analysis of prey remains (hair and hard parts).

Importantly, the lack of wader DNA in our sampled scats does not rule out indirect predator impacts. Elevated predator presence may deter nesting or foraging; affecting wader fecundity and recruitment or lead to site abandonment. While this study focused on mammalian predators, avian generalists (e.g., corvids, buzzards), often associated with forestry (Hughes et al. 2024), may also exert direct and indirect pressure. Further research, such as diet analysis of avian predators, predator removal studies, or metabarcoding of saliva from artificial eggs, could improve understanding of predation impacts; though these approaches also face species‐specific limitations.

## 5 Conservation Implications

DNA metabarcoding of scats presents a way to gain an improved understanding of interspecific relationships inferred from correlative data. Our findings also demonstrate that in scat studies where genetic analysis cannot be used to confirm predator identity, the potential for misidentification to lead to incorrect assumptions exists. Our study challenges the assumption that mammalian predators associated with non‐native forestry predate waders in adjacent peatland in the Flow Country. However, it does not discount the aforementioned indirect effects of their presence. The results support a ‘coexistence conservation’ approach (Evans et al. 2022), suggesting that managing wader responses to predator‐associated habitats may be more effective at conserving waders than directly targeting predators using costly or controversial control measures. Ultimately, conservation efforts are likely to benefit from increasing direct monitoring to further our understanding of their complex trophic and non‐trophic interactions that could affect vulnerable prey species as habitats undergo increasing alteration and new predators move in as part of natural (habitat change associated) and artificial (rewilding‐facilitated) range expansions.

## Author Contributions

**R. McHenry:** conceptualization (lead), data curation (lead), formal analysis (lead), funding acquisition (supporting), investigation (lead), methodology (lead), project administration (lead), resources (equal), software (supporting), validation (equal), visualization (lead), writing – original draft (lead), writing – review and editing (equal). **L. J. Mitchell:** conceptualization (supporting), data curation (supporting), formal analysis (supporting), methodology (equal), resources (equal), software (equal), supervision (supporting), validation (equal), visualization (supporting), writing – review and editing (equal). **J. Smart:** supervision (supporting), writing – review and editing (supporting). **R. Andersen:** conceptualization (supporting), formal analysis (supporting), funding acquisition (lead), investigation (supporting), methodology (supporting), project administration (equal), resources (equal), supervision (lead), visualization (supporting), writing – review and editing (equal).

## Conflicts of Interest

The authors declare no conflicts of interest.

## Supporting information


**Appendix S1:** ece371961‐sup‐0001‐Appendix.docx.

**Data S1:** Frequency of occurence PM and Fox.

**Data S2:** RRA table PM and Fox.

**Data S3:** Seq results_copy1.

## Data Availability

All data referred to in this manuscript is available as Supporting Information included for publication. Please contact Rob McHenry by email robmchenry@protonmail.com for any additional data inquiries.
