# Supplementary material for: Scat‐Tered Evidence. Understanding the Diet of Forest‐Associated Mammalian Mesopredators in a UK Peatland Ecosystem
Source: Ecol Evol. 2025 Aug 14;15(8):e71961. doi: 10.1002/ece3.71961 (PMC12354625; doi:10.1002/ece3.71961)
Supplement: Supplementary file 1 — Appendix S1: ece371961‐sup‐0001‐Appendix.docx. [file ECE3-15-e71961-s001.docx]

**Appendix**

**12S Kelly Riaz vertebrate metabarcoding – rough protocol**

Libraries were prepared for sequencing using a nested metabarcoding workflow with a two-step PCR protocol, where Multiplex Identification (MID) tags (unique 8-nucleotide sequences) were included in the first and second PCR for sample identification (Kitson et al. 2019). DNA extracts were PCR-amplified using vertebrate-specific primers that target a 97 bp fragment of the mitochondrial 12S ribosomal RNA (rRNA) region in fish (Riaz et al. 2011). Primers were tagged for the present study to include MID tags, heterogeneity spacers, sequencing primers, and pre-adapters. There were 24 unique MID tags for the forward and 24 unique MID tags for the reverse primers. This allowed 24 samples to each be labelled with a unique forward and a unique reverse primer to reduce barcode misassignment and tag jumps (Deakin et al. 2014; Schnell et al. 2015). During the first PCR, samples were processed in batches (i.e. up to 22 eDNA samples, one negative PCR control and one PCR positive control). The PCR positive control was black tilapia (Oreochromis niloticus) DNA (0.025 ng/μl). Black tilapia is tropical aquaculture fish not found in UK freshwater habitats.

The first PCR was performed in triplicate for each sample/control to combat stochasticity arising from low target DNA concentrations. PCR replicates for each sample/control had the same tag combination. 96 well plate with 8-strip domed caps were used for PCR reactions. PCR reactions were performed in 15 μl volumes, consisting of: 7.5 μl of Q5® High-Fidelity 2x Master Mix (New England Biolabs), 0.3 μl of Thermo Scientific Bovine Serum Albumin (Fisher Scientific UK Ltd.), 3.7 μl of nuclease free water (Fisher Scientific UK Ltd.), 1.8 μl of each 10 μM tagged primer, and 1.7 μl of template DNA. PCR was performed on an Agilent Surecycler 8800 thermal cycler (Agilent Technologies UK) with the following thermocycling profile: 98°C for 5 mins, 35 cycles of 98°C for 10 s, 58°C for 20 s and 72°C for 30 s, 72°C for 7 mins then held at 4°C.

PCR products were stored at 4°C until PCR technical replicates for each sample/control were pooled. 2 μl of each pooled PCR product was visualised using Zero Agarose Gel instrument (ZAG, Agilent Technologies) using 1-1500 bp gel kit. PCR products were deemed positive where there was amplification at the expected size (220-260 bp) on the gel. PCR products were stored at 4°C until they were pooled according to band strength/ concentration obtained from gel image on the ZAG (no band /very faint band = 20 μl, faint band = 15 μl, bright band = 10 μl, very bright band = 5 μl) to create sub-libraries for a size selection bead purification protocol. Ratio of 1.5x Promega ProNex magnetic beads (Promega UK) to 50 μl of each sub-library was used for purification. 2uL of the eluted DNA (from the total elution volume 25 μl) was visualised on Zero Agarose Gel (Agilent Technologies UK) using 1-1500 bp gel kit and then was stored at 4°C until second PCR amplification.

The second PCR bound pre-adapters, MID tags, and Illumina adapters to the purified sub-libraries. 3 unique forward and reverse MID tag combinations were selected and applied to 3 the sub-libraries. Two replicates were performed for each sub-library in 50 μl volumes, consisting of: 25 μl of Q5 High-Fidelity 2x Master Mix (New England Biolabs), 15 μl of nuclease free water (Fisher Scientific UK Ltd.), 6 μl of each 10 μM tagged primer (final concentration 0.6 μM; Thermo Scientific UK Ltd.), and 4 μl of template DNA. PCR was performed on Agilent Surecycler 8800 thermal cycler (Agilent Technologies UK) with the following thermocycling profile: 95°C for 3 mins, 10 cycles of 98°C for 20 s and 72°C for 1 min, 72°C for 5 mins then held at 4°C. PCR duplicates for each sub-library had the same tag combination.

PCR products were stored at 4°C until duplicates for each sub-library were pooled. 2 μl of each pooled PCR product was visualised on Zero Agarose Gel (ZAG, Agilent Technologies, UK) using 1-1500 bp gel kit. PCR products were deemed positive where there was amplification at the expected size (300-330 bp) on the gel. Sub-libraries were stored at 4°C until double-size selection bead purification. Ratios of 1.2x and 0.4x ProNex magnetic beads (Promega UK) to 50 μl of each sub-library were used for purification. 2uL of the eluted DNA (total elution volume 50 μl) was visualised on Zero Agarose Gel (Agilent Technologies UK) using 1-1500 bp gel kit and then was stored at 4°C until final normalisation.

Sub-libraries were quantified on a Qubit 3.0 fluorometer using a dsDNA HS Assay Kit (Invitrogen) and normalised by pooling according to sample size and library concentration. Based on the Qubit™ concentration, the library was diluted to 6 nM. The library was then quantified by qPCR using the Kapa Library Quant Kit for Illumina (Roche Sequencing UK). Based on the qPCR concentration, the library concentration was adjusted to 4 nM and denatured following the Illumina MiSeq library denaturation and dilution guide. The final library was sequenced at 13 pM with 10% PhiX Control on an Illumina MiSeq using 2 x 300 bp V3 chemistry (Illumina).

#### Vertebrate bioinformatics

Sequencing data was automatically demultiplexed to separate (forward and reverse) fastq files per library using the onboard Illumina MiSeq Reporter software. Library sequence reads were further demultiplexed to sample using a custom Python script. *Tapirs*, a reproducible workflow for the analysis of DNA metabarcoding data (<https://github.com/EvoHull/Tapirs>), was used for taxonomic assignment of demultiplexed sequencing reads. *Tapirs* uses the *Snakemake* workflow manager (Köster and Rahmann 2012) and a *conda* virtual environment to ensure software compatibility.

Raw reads were quality trimmed from the tail with a 5 bp sliding window (qualifying phred score of Q30 and an average window phred score of Q30) using *fastp* (Chen et al. 2018), allowing no more than 40% of the final trimmed read bases to be below Q30. Primers were removed by trimming the first 18 bp of both forward and reverse reads. Reads were then tail cropped to a maximum length of 106 bp and reads shorter than 90 bp were discarded.

Sequence read pairs were merged into single reads using *fastp*, provided there was a minimum overlap of 20 bp, no more than 5% mismatches and no more than 5 mismatched bases between pairs. Only forward reads were kept from read pairs that failed to be merged. A final length filter removed any reads longer than 110 bp to ensure sequence lengths approximated the expected fragment size (~97 bp).

Redundant sequences were removed by clustering at 100% read identity and length (--derep_fulllength) in *VSEARCH* (Rognes et al. 2016). Clusters represented by less than three sequences were omitted from further processing. Reads were further clustered (--cluster_unoise) to remove redundancies due to sequencing errors (retaining all cluster sizes). Retained sequences were screened for chimeric sequences with *VSEARCH* (--uchime3_denovo).

The final clustered, non-redundant query sequences were then compared against a curated UK vertebrate reference database (Harper et al. 2018) using BLAST (Zhang et al. 2000). Taxonomic identity was assigned using a custom majority lowest common ancestor (MLCA) approach based on the top 2% query BLAST hit bit-scores, with at least 90% query coverage and a minimum identity of 98%. Of these filtered hits, 80% of unique taxonomic lineages therein had to agree at descending taxonomic rank (domain, phylum, class, order, family, genus, species) for it to be assigned a taxonomic identity. If a query had a single BLAST hit it was assigned directly to this taxon only if it met all MLCA criteria. Read counts assigned to each taxonomic identity were calculated from query cluster sizes. Lowest taxonomic rank was to species and assignments higher than order were classed as unassigned. Following taxonomic assignment a noise threshold of 0.1% of total reads per sample was applied to remove low frequency reads (Hänfling et al. 2016). Most reads were assigned to the species level, but as the molecular marker used here cannot distinguish certain species reliably, the reads belonging to these species were assigned to the next possible higher taxonomic level. Reads assigned to positive controls, reads which could not be assigned to any taxon and samples with no taxonomically assignable reads were also removed from the data set.
